# Interaction of myricetin, ampelopsin (dihydromyricetin), and their sulfate metabolites with serum albumin, cytochrome P450 (CYP2C9, 2C19, and 3A4) enzymes, and organic anion‐transporting polypeptides (OATP1B1 and OATP2B1)

**DOI:** 10.1002/prp2.70021

**Published:** 2024-09-29

**Authors:** Ágnes Dombi, Hana Kaci, Kateřina Valentová, Éva Bakos, Csilla Özvegy‐Laczka, Miklós Poór

**Affiliations:** ^1^ Department of Pharmacology, Faculty of Pharmacy University of Pécs Pécs Hungary; ^2^ Drug Resistance Research Group, Institute of Molecular Life Sciences Research Centre for Natural Sciences, HUN‐REN Budapest Hungary; ^3^ Doctoral School of Biology, Institute of Biology Eötvös Loránd University Budapest Hungary; ^4^ Institute of Microbiology of the Czech Academy of Sciences Prague Czech Republic; ^5^ Department of Laboratory Medicine, Medical School University of Pécs Pécs Hungary; ^6^ Molecular Medicine Research Group, János Szentágothai Research Centre University of Pécs Pécs Hungary

**Keywords:** myricetin, ampelopsin, human serum albumin, CYP enzymes, OATP transporters

## Abstract

Myricetin (MYR) and ampelopsin (AMP, or dihydromyricetin) are flavonoid aglycones found in certain plants and dietary supplements. During the presystemic biotransformation of flavonoids, mainly sulfate and glucuronide derivatives are produced, which are the dominant metabolites in the circulation. In this study, we tested the interactions of MYR, myricetin‐3′‐*O*‐sulfate (M3′S), AMP, and ampelopsin‐4′‐*O*‐sulfate (A4′S) with human serum albumin (HSA), cytochrome P450 enzymes (CYPs), and organic anion‐transporting polypeptides (OATPs) using in vitro models, including the recently developed method for measuring flavonoid levels in living cells. M3′S and MYR bound to albumin with high affinity, and they showed moderate displacing effects versus the Site I marker warfarin. MYR, M3′S, AMP, and A4′S exerted no or only minor inhibitory effects on CYP2C9, CYP2C19, and CYP3A4 enzymes. M3′S and MYR caused considerable inhibitory actions on OATP1B1 at low micromolar concentrations (IC_50_ = 1.7 and 6.4 μM, respectively), while even their nanomolar levels resulted in strong inhibitory effects on OATP2B1 (IC_50_ = 0.3 and 0.4 μM, respectively). In addition, M3′S proved to be a substrate of OATP1B1 and OATP2B1. These results suggest that MYR‐containing dietary supplements may affect the OATP‐mediated transport of certain drugs, and OATPs are involved in the tissue uptake of M3′S.

## Graphical abstract

Myricetin and myricetin‐3′‐O‐sulfate proved to be strong inhibitors of organic anion transporting polypeptides (OATPs) at low micromolar (OATP1B1) and at nanomolar (OATP2B1) concentrations.

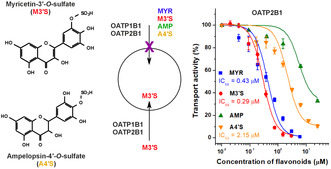

**Abbreviations**
A4′S: ampelopsin‐4′‐*O*‐sulfate
AMP: ampelopsin
APB: 2‐aminoethyl diphenylborinate
BSA: bovine serum albumin
BSP: bromosulfophthalein
CYP: cytochrome P450
HSA: human serum albumin
M3′S: myricetin‐3′‐*O*‐sulfate
MYR: myricetin
OATP: organic anion‐transporting polypeptide

## 1 INTRODUCTION

**FIGURE 1:**
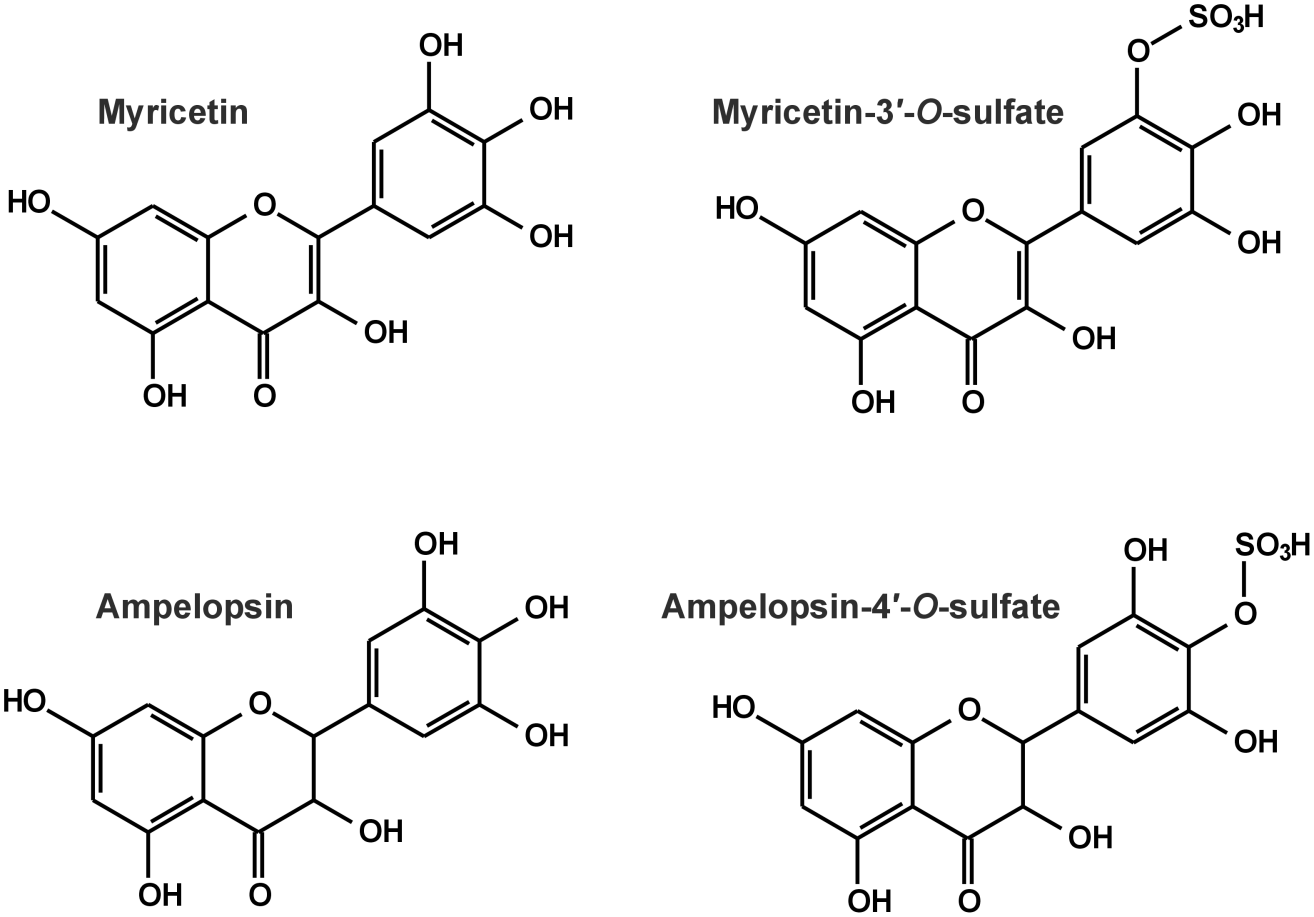
Chemical structures of myricetin (MYR), myricetin‐3′‐*O*‐sulfate (M3′S), ampelopsin (AMP), and ampelopsin‐4′‐*O*‐sulfate (A4′S).

Myricetin (MYR; Figure 1) is a flavonol aglycone. MYR and its glycosides are found in several foodstuffs, including fruits, vegetables, honey, red wine, and tea^1^; and the aglycone is also contained by dietary supplements (typical recommended daily dose: 100 mg).^2^ MYR may have beneficial health effects, and its potential anti‐inflammatory, antidiabetic, immunomodulatory, antimicrobial, and antitumor impacts have been reported.^1^, ^3^ Like other flavonoids, MYR has low oral bioavailability (less than 10% in rats).^4^ The peak plasma concentrations of MYR and its (sulfate and glucuronide) metabolites were together 8 μM after the per os treatment of rats with 100 mg/kg of MYR.^4^

Ampelopsin (AMP, or dihydromyricetin; Figure 1) is a flavanonol aglycone. AMP is contained at very high levels (20–30 w/w%) in *Ampelopsis grossedentata*, which is widely applied as vine tea in Chinese traditional medicine, but it is also found in other medicinal plants.^5^ Furthermore, AMP is the active ingredient of dietary supplements (typical suggested daily dose: 300–600 mg).^2^ Based on earlier studies, AMP may have cardioprotective, antihypertensive, anti‐inflammatory, antidiabetic, hepatoprotective, neuroprotective, and antitumor effects.^6^ In a double‐blind randomized controlled clinical trial, AMP improved glucose and lipid metabolism and exerted anti‐inflammatory action in patients who suffer from nonalcoholic fatty liver disease.^7^ Based on rat studies, AMP has low oral bioavailability (approximately 4%); glucuronidation, sulfation, dehydroxylation, and methylation are its major biotransformation pathways.^8^, ^9^ After the peroral administration of 100 mg/kg AMP, approximately 300–500 nM peak plasma concentrations of the aglycone were detected in rats.^10^, ^11^

Human serum albumin (HSA) interacts with many endogenous compounds, drugs, and toxins in human circulation; the formation of highly stable ligand‐HSA complexes can influence the pharmacokinetic properties of the bound ligand molecules.^12^, ^13^ Sudlow's Site I and Sudlow's Site II are the most important drug‐binding regions on HSA.^13^ Earlier reports demonstrated that MYR and AMP form stable complexes with HSA occupying Site I as their high‐affinity binding site.^14^, ^15^ In previous studies, the sulfate metabolites of certain flavonoids bound to HSA with higher affinity than the parent aglycones, and caused stronger displacement of the Site I marker warfarin.^16^, ^17^, ^18^

Cytochrome P450 (CYP) enzymes (including CYP2C9, 2C19, and 3A4) are key players in the oxidative biotransformation of drugs and other xenobiotics.^19^, ^20^ Previous studies suggest that MYR and AMP can inhibit certain CYPs (e.g., CYP3A4), while we found no data regarding their sulfate metabolites.^21^, ^22^, ^23^, ^24^

Organic anion‐transporting polypeptides (OATPs) are solute carrier (SLC)‐type membrane transporters mediating the tissue uptake of endo‐ and exobiotics, including drugs.^25^ OATP1B1 plays a major role in the hepatic uptake of drugs, while OATP2B1 is mainly located in enterocytes and blood–brain barrier endothelial cells.^26^, ^27^ Earlier reports suggest moderate inhibitory effects of MYR and AMP on OATP1B1.^28^, ^29^, ^30^, ^31^ Furthermore, MYR only weakly inhibited OATP2B1 activity.^32^ Nevertheless, sulfate conjugation may enhance the inhibitory action of flavonoids on OATPs.^16^, ^33^, ^34^ In addition, certain flavonoid metabolites are not only inhibitors of OATPs but they are also transported substrates of these carriers: Recently, we demonstrated the OATP‐mediated cellular uptake of quercetin and luteolin conjugates, applying their fluorescence detection in the presence of 2‐aminoethyl diphenylborinate (APB).^35^

In this study, we aimed to investigate the interactions of MYR, myricetin‐3′‐*O*‐sulfate (M3′S; Figure 1), AMP, and ampelopsin‐4′‐*O*‐sulfate (A4′S; Figure 1) with HSA, CYP enzymes, and OATP transporters. The complex formation of the flavonoids with albumin was evaluated based on fluorescence quenching studies. To examine the displacing ability of MYR, AMP, and their sulfate conjugates versus the Site I marker warfarin, ultrafiltration experiments were performed. Inhibitory effects of flavonoids on CYP2C9, 2C19, and 3A4 were tested in vitro using human recombinant enzymes. Finally, the interaction of MYR, M3′S, AMP, and A4′S with OATP1B1 and OATP2B1 was examined on OATP‐overexpressing cell lines applying both indirect and direct assays.

## 2 MATERIALS AND METHODS

### 2.1 Reagents

MYR and AMP were purchased from abcr GmbH (Karlsruhe, Germany) and Herb Nutritionals Ltd. (Shanghai, China), respectively. Myricetin‐3′‐*O*‐sulfate (M3′S) and ampelopsin‐4′‐*O*‐sulfate (A4′S) were synthesized chemo‐enzymatically as it has been described.^36^ Diclofenac, 4′‐hydroxydiclofenac, sulfaphenazole, (*S*)‐mephenytoin, and 4‐hydroxymephenytoin were from Carbosynth (Berkshire, UK). CypExpress™ Cytochrome P450 (CYP2C9, 2C19, and 3A4) human kits, ticlopidine, testosterone, 6β‐hydroxytestosterone, ketoconazole, racemic warfarin, HSA (product code: A1653), bovine serum albumin (BSA), disulfopyrene, pyranine, 2‐aminoethyl diphenylborinate (APB), bromosulfophthalein (BSP), and further chemicals (if not stated otherwise) were obtained from Merck (Darmstadt, Germany). Stock solutions of flavonoids (10 mM) were prepared in dimethyl sulfoxide (DMSO, spectroscopic grade; Fluka, Charlotte, NC, US) and stored at −20°C. APB was dissolved in DMSO (125 mg/mL) and stored at −20°C. BSP solution (10 mM) was prepared in distilled water.

### 2.2 Spectroscopic studies

Fluorescence spectroscopic measurements were performed in phosphate‐buffered saline (PBS, pH 7.4) at room temperature, using a Hitachi F‐4500 spectrofluorometer (Tokyo, Japan). Increasing amounts of flavonoids (final concentrations: 0, 1, 2, 3, 4, 5, and 6 μM) were added to HSA (2 μM), after which fluorescence emission spectra were collected (*λ*_ex_ = 295 nm). UV–Vis spectra of MYR, M3′S, AMP, and A4′S were also recorded, and their inner‐filter effects were corrected as it has been reported.^37^, ^38^ Flavonoid–HSA interactions were evaluated based on the graphical application of the Stern–Volmer equation^18^, ^39^:

(1) $$\frac{I_{0}}{I}=1+K_{\mathrm{SV}}\times \left[Q\right]$$

where *K*_SV_ is the Stern–Volmer quenching constant, *I*_0_ denotes the emission intensity of HSA alone at 340 nm, *I* marks the emission signal of albumin at 340 nm in the presence of flavonoids, while [Q] is the molar concentration of the quencher. Thereafter, binding constants (*K*) of the formed complexes were determined based on the modified Stern–Volmer equation^39^, ^40^:

(2) $$\frac{I_{0}}{\left(I_{0}-I\right)}=\frac{1}{f_{a}\times K}\times \frac{1}{\left[Q\right]}+\frac{1}{f_{a}}$$

where *f*_a_ is the fraction of the accessible fluorophore.

Using the binding/association constants and assuming 1:1 stoichiometry of complex formation, we estimated the bound fractions of M3′S and A4′S in the circulation based on the following equation^41^:

(3) $$K=\frac{\left[\mathrm{FH}\right]}{\left[F\right]\times \left[\mathrm{HSA}\right]}$$

where [*F*], [HSA], and [FH] are the molar concentrations of the unbound free flavonoid, the unbound free HSA, and the flavonoid–HSA complex, respectively. In these calculations, we hypothesized the presence of 1.0 μM levels of M3′S or A4′S in the presence of 600 μM (≈ 40 g/L) of HSA.

### 2.3 Ultrafiltration studies

The impacts of MYR, M3′S, AMP, and A4′S on the albumin‐bound fraction of the Site I marker warfarin were examined with the previously reported ultrafiltration method.^17^, ^18^ Briefly, samples contained warfarin (1 μM) and HSA (5 μM) without or with flavonoids (20 μM) in PBS (pH 7.4). After two washing steps of the filters (Amicon Ultra‐0.5 Centrifugal Filter Units, 30 kDa molecular weight cut‐off; Merck, Darmstadt, Germany) with water then PBS (both 500 μL), samples (500 μL) were centrifuged for 10 min (7500 g, 25°C, fixed angle rotor).

Warfarin was quantified using an HPLC system (Jasco, Tokyo, Japan) with an autosampler (AS‐4050), a binary pump (PU‐4180), a fluorescence detector (Jasco FP‐920), and the ChromNAV2 software. The concentrations of warfarin in the filtrate were directly determined by applying the previously described method.^17^, ^18^ Briefly, samples (20 μL) were driven through a pre‐column (SecurityGuard C18, 4.0 × 3.0 mm; Phenomenex, Torrance, CA, USA) linked to a Nova‐Pak C18 (150 × 3.9 mm, 4 μm; Waters, Milford, MA, USA) analytical column with 1.0 mL/min flow rate at room temperature, using sodium phosphate buffer (20 mM, pH 7.0), methanol, and acetonitrile (70:25:5 v/v%) in the mobile phase. The fluorescence detection of warfarin was carried out at 310 and 390 nm excitation and emission wavelengths, respectively.

### 2.4 CYP assays

To test the inhibitory effects of MYR, M3′S, AMP, and A4′S on CYP enzymes, CypExpress Cytochrome P450 human kits were applied, using U.S. Food and Drug Administration (FDA)‐recommended substrates and positive controls. CYP2C9 (diclofenac hydroxylation), 2C19 ((*S*)‐mephenytoin hydroxylation), and 3A4 (testosterone hydroxylation) assays were carried out as it has been described, without modifications.^33^, ^38^

Substrates and metabolites were analyzed using the HPLC system described in 2.3, except a UV detector (UV‐975; Jasco) was applied. To quantify diclofenac and 4′‐hydroxydiclofenac, (*S*)‐mephenytoin and 4‐hydroxymephenytoin, and testosterone and 6β‐hydroxytestosterone, our previously reported HPLC methods were applied (see the brief descriptions below).^16^

In the CYP2C9 assay, the samples (each 20 μL) were driven through a pre‐column (SecurityGuard C8, 4.0 × 3.0 mm; Phenomenex) linked to a Mediterranea Sea8 (C8, 150 × 4.6 mm, 5 μm; Teknokroma, Barcelona, Spain) analytical column with 1.0 mL/min flow rate at room temperature. The isocratic elution was carried out with phosphoric acid (6 mM) and acetonitrile (48:52 v/v%) as the mobile phase. Diclofenac and 4′‐hydroxydiclofenac were detected at 275 nm.

In the CYP2C19 assay, the samples (each 20 μL) were driven through a pre‐column (SecurityGuard C8, 4.0 × 3.0 mm; Phenomenex) linked to a Luna (C8, 150 × 4.6 mm, 5 μm; Phenomenex) analytical column with 1.0 mL/min flow rate at room temperature. The isocratic elution was carried out with sodium acetate buffer (6.9 mM, pH 4) and acetonitrile (72:28 v/v%) as the mobile phase. (*S*)‐Mephenytoin and 4‐hydroxymephenytoin were detected at 230 nm.

In the CYP3A4 assay, the samples (each 20 μL) were driven through a pre‐column (SecurityGuard C18, 4.0 × 3.0 mm; Phenomenex) linked to Kinetex EVO‐C18 (C18, 150 × 4.6 mm, 5 μm; Phenomenex) analytical column with 1.2 mL/min flow rate at room temperature. The isocratic elution was carried out with methanol, water, and acetic acid (53:46:1 v/v%) as the mobile phase. Testosterone and 6β‐hydroxytestosterone were detected at 240 nm.

### 2.5 Cell cultures

Human A431 cells overexpressing OATPs (OATP1B1 or OATP2B1) and their mock‐transfected controls were established earlier.^42^, ^43^ The cells were maintained at 37°C with 5% CO_2_ in Dulbecco's Modified Eagle Medium (DMEM; Thermo Fischer Scientific, Waltham, MA, USA) supplemented with fetal bovine serum (10%), l‐glutamine (2 mM), penicillin (100 units/mL) and streptomycin (100 μg/mL).

### 2.6 Testing the inhibitory effects of flavonoids on OATP transporters

The inhibitory impacts of MYR, AMP, and their sulfate metabolites on OATP1B1 and OATP2B1 were examined using the fluorescent dye substrates disulfopyrene (6,8‐dihydroxy‐1,3‐disulfopyrene) and pyranine, respectively.^42^, ^43^ The day before the transport measurements, A431 cells overexpressing OATP1B1 or OATP2B1 (and their mock‐transfected controls) were seeded onto 96‐well plates at a density of 80 000 cells/well in DMEM (200 μL). Next day, the medium was removed, the cells were washed three times with PBS (pH 7.4, 200 μL) at room temperature, then the cells were pre‐incubated with 50 μL of Hank's Balanced Salt Solution (HBSS, pH 7.4; OATP1B1) or uptake buffer (pH 5.5; OATP2B1) for 5 min at 37°C with or without the flavonoids.^43^ The transport reaction was started by adding 50 μL of disulfopyrene‐containing (final concentration: 10 μM) HBSS (OATP1B1 assay), or pyranine‐containing (final concentration: 20 μM) uptake buffer (OATP2B1 assay). After 10 min (OATP1B1) or 15 min (OATP2B1), the transport was stopped by removing the supernatant and washing the cells three times with ice‐cold PBS (200 μL). Then the fluorescence was measured in 200 μL of PBS using an Enspire plate reader (PerkinElmer, Waltham, MA, US) at excitation and emission wavelengths of 460/510 nm. OATP‐dependent transport was determined by subtracting the fluorescence detected in mock control cells from that of OATP‐expressing cells. Transport activity was measured based on the fluorescence signal in the absence of flavonoids (100%). IC_50_ values were calculated by sigmoidal fitting (Hill1), using the Origin software (version 2018, OriginLab Corporation, Northampton, MA, USA).

### 2.7 Fluorescence spectra of flavonoid‐APB complexes in a cell‐free environment

To examine the fluorescence spectra of the flavonoids labeled with APB, samples (200 μL) contained the flavonoid (25 pmol), APB (250 μg/mL), and BSA (1 mg/mL) in PBS.^35^ We used an Enspire plate reader to measure the fluorescence spectra of the flavonoids. The excitation and emission wavelength ranges were set to 400–500 nm and 500–700 nm, respectively. We observed the following excitation and emission maxima: 480/550 nm for MYR, 480/540 nm for M3′S, and 460/540 nm for AMP and A4′S.

### 2.8 Uptake of flavonoids by OATP‐overexpressing versus mock cells

Direct uptake of AMP, MYR, and their sulfate metabolites were examined in A431 cells overexpressing OATP1B1 or OATP2B1, and in their mock‐transfected controls using APB as a fluorescence enhancer. One day before the uptake measurements, the cells were seeded onto 96‐well plates at a density of 80 000 cells/well in DMEM (200 μL). The following day, the cell culture medium was removed, and the cells were rinsed thrice with PBS (200 μL). Thereafter, the cells were preincubated with HBSS (50 μL) for 5 min at 37°C. The uptake reaction was initiated by the addition of a further 50 μL of HBSS containing AMP, MYR, or their sulfate conjugates (10 μM). After incubating for 15 min at 37°C, the reaction was stopped by removing the supernatant and rinsing the cells three times with ice‐cold PBS. Then PBS buffer (200 μL) containing APB (250 μg/mL) and BSA (1 mg/mL) was added to each well.^35^ The fluorescence was read using an Enspire plate reader using the wavelengths listed in Section 2.7.

The concentration‐dependent uptake of M3′S was also tested under the same conditions, except the cells were incubated with 0–10 μM M3′S for 2 min (OATP1B1) or 5 min (OATP2B1).

To test the effect of BSP (a known inhibitor of OATPs^44^) on the cellular uptake of M3′S, cells were preincubated with HBSS buffer (50 μL) with or without BSP (20 μM) for 5 min at 37°C. Thereafter, we added 50 μL of HBSS containing M3′S (final concentration: 2 μM), and cells were further incubated at 37°C for 2 min (OATP1B1) or 5 min (OATP2B1). The reaction was stopped by removing the supernatant. After washing the cells with ice‐cold PBS, a PBS buffer with APB/BSA was added. Fluorescence was determined by applying an Enspire plate reader at excitation/emission wavelengths of 480/540 nm.

### 2.9 Statistical analyses

Means and standard error of the mean (± SEM) values demonstrated are at least from three independent experiments. Statistical differences (*p* < .05 and *p* < .01) were evaluated using one‐way ANOVA with Tukey post‐hoc test (SPSS Statistics software, IBM, Armonk, NY, USA).

### 2.10 Nomenclature of targets and ligands

Key protein targets and ligands in this article are hyperlinked to corresponding entries in http://www.guidetopharmacology.org, the common portal for data from the IUPHAR/BPS Guide to PHARMACOLOGY,^45^ and are permanently archived in the Concise Guide to PHARMACOLOGY 2023/24.^46^, ^47^

## 3 RESULTS

### 3.1 Interaction of MYR, M3′S, AMP, and A4′S with human serum albumin

**FIGURE 2:**
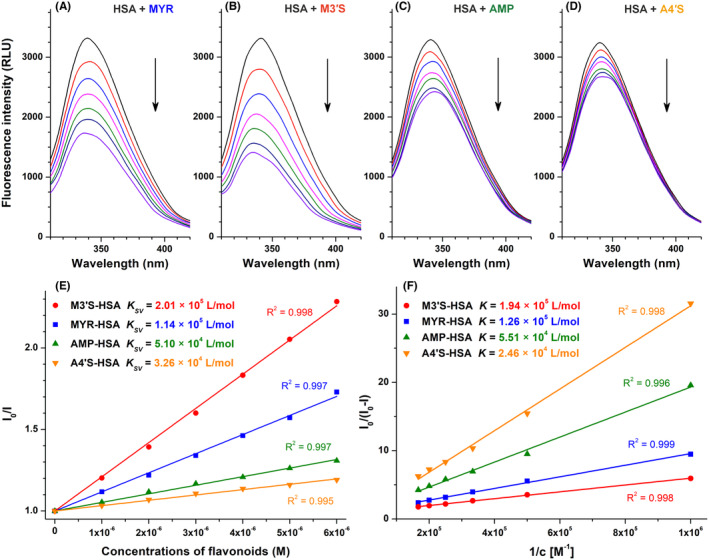
Representative fluorescence emission spectra of HSA (2 μM) in the presence of increasing concentrations (0–6 μM) of MYR (A), M3′S (B), AMP (C), and A4′S (D) in PBS (pH 7.4). Stern–Volmer plots (E) and modified Stern–Volmer plots (F) of flavonoid‐HSA complexes (λ_ex_ = 295 nm, λ_em_ = 340 nm).

To test the potential interactions of MYR, M3′S, AMP, and A4′S with HSA, fluorescence quenching studies were performed, where the emission spectra of the protein were collected in the presence of increasing flavonoid concentrations. Even after the correction of their inner‐filter effects, MYR (Figure 2A), M3′S (Figure 2B), AMP (Figure 2C), and A4′S (Figure 2D) considerably reduced the emission intensity of HSA at 340 nm in a concentration‐dependent manner. The largest quenching was induced by M3′S, followed by MYR, AMP, and A4′S. Stern–Volmer (Figure 2E) and modified Stern–Volmer (Figure 2F) plots of flavonoid–albumin complexes showed good linearity (R^2^ > .99). *K*_*SV*_ values and binding constants were in good agreement (Figure 2), representing the formation of stable MYR–HSA (*K* = 1.3 × 10^5^ L/mol) and M3′S–HSA (*K* = 1.9 × 10^5^ L/mol), while moderately stable AMP–HSA (*K* = 5.5 × 10^4^ L/mol) and A4′S–HSA (*K* = 2.5 × 10^4^ L/mol) complexes. Based on the binding/association constants and assuming 1:1 stoichiometry of complex formation, if we estimate 1 μM plasma levels of M3′S and A4′S in the presence of 600 μM HSA (≈ 40 g/L), then the albumin‐bound fraction of M3′S exceeds 99% and the bound fraction of A4′S approximates 94%.

**FIGURE 3:**
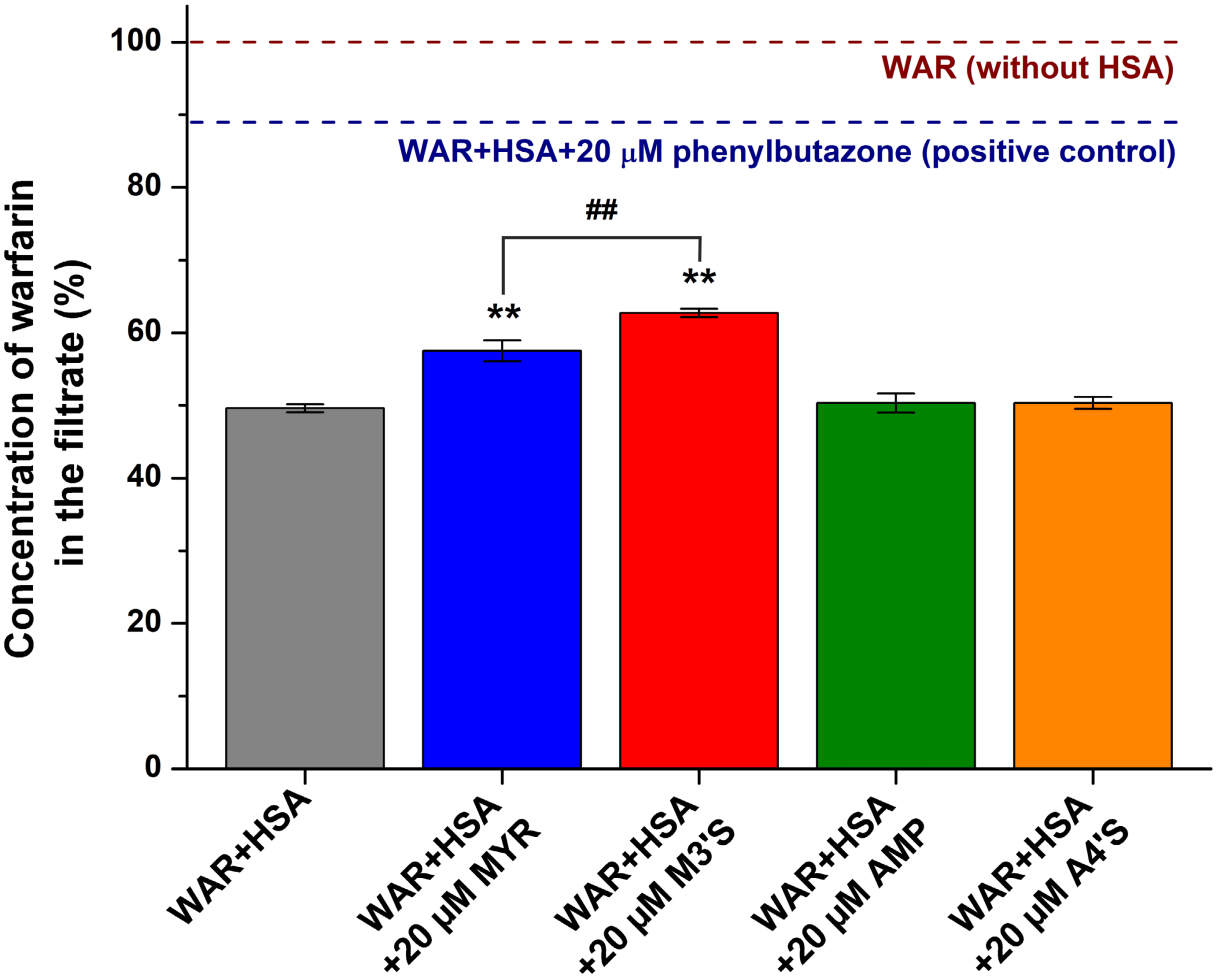
Effects of MYR, M3′S, AMP, and A4′S on the filtered fraction of warfarin. Samples, containing warfarin (1.0 μM) and HSA (5.0 μM) in the absence and presence of flavonoids (20 μM) in PBS (pH 7.4), were filtered through the filter units (molecular weight cut‐off = 30 kDa; **p < .01: compared to WAR+HSA; ^##^ *p* < .01: effects of M3′S compared to MYR). Warfarin (1.0 μM) filtered without HSA (100%; brown) and the impact of the positive control phenylbutazone (20 μM; navy blue) were marked with dashed lines.

After quenching studies, ultrafiltration experiments were also performed to examine the potential ability of flavonoids to displace the Site I marker warfarin from albumin. Importantly, HSA and albumin‐bound warfarin are not able to pass through the filter unit applied. In the filtrate, warfarin levels were not affected by AMP and A4′S; however, MYR and M3′S caused statistically significant (*p* < .01) but only moderate increases in the filtered fraction of the site marker (Figure 3). In addition, M3′S caused a stronger (*p* < .01) impact compared to its parent aglycone.

### 3.2 Interaction of MYR, M3′S, AMP, and A4′S with CYP enzymes

**FIGURE 4:**
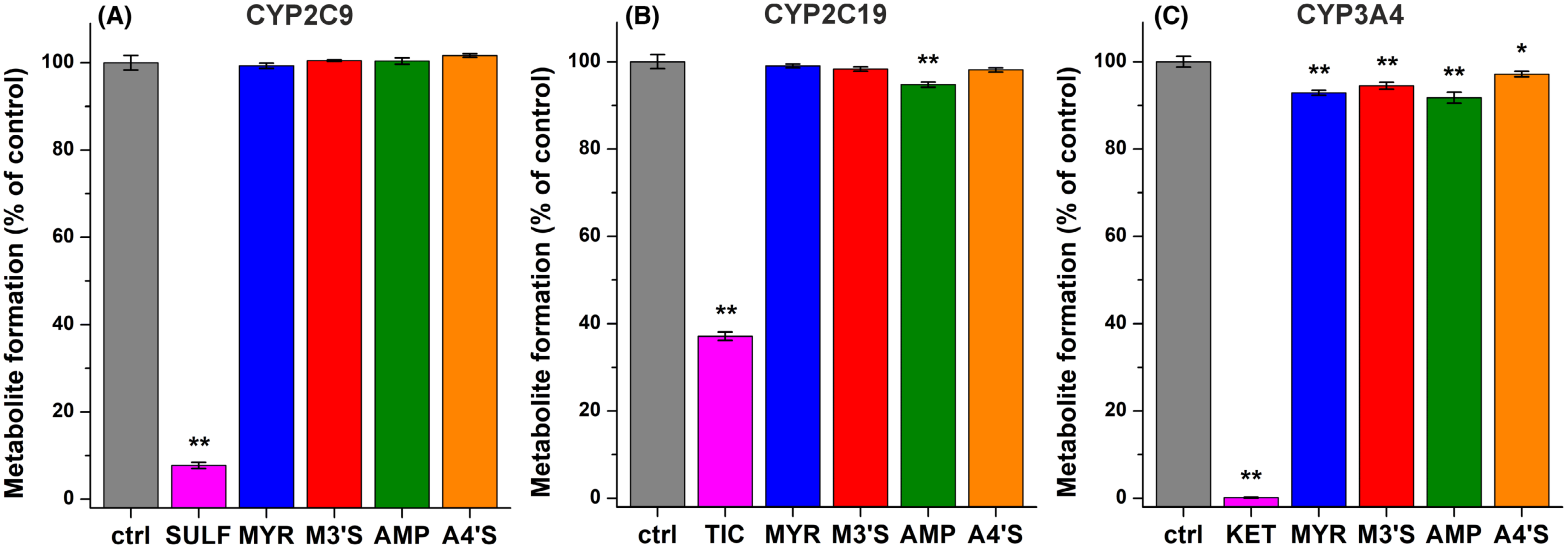
Effects of MYR (blue), M3′S (red), AMP (green), and A4′S (orange) on CYP2C9 (A), CYP2C19 (B), and CYP3A4 (C) enzymes. Metabolite formation (% of control ± SEM) was examined in the absence and presence of the positive control inhibitors (20 μM; magenta) or flavonoids (20 μM) regarding CYP2C9‐catalyzed diclofenac hydroxylation, CYP2C19‐catalyzed (*S*)‐mephenytoin hydroxylation, and CYP3A4‐catalyzed testosterone hydroxylation (*n* = 3; substrate concentrations = 5 μM; *p < .05, **p < .01; SULF, sulfaphenazole; TIC, ticlopidine; KET, ketoconazole).

The potential inhibitory effects of MYR, M3′S, AMP, and A4′S on CYP2C9, 2C19, and 3A4 enzymes were also examined. In each assay, we applied 20 μM flavonoid versus 5 μM substrate concentrations. In the corresponding assays, the FDA‐recommended inhibitors (sulfaphenazole, ticlopidine, and ketoconazole) caused marked decreases in metabolite formation (Figure 4). DMSO levels were uniformly 0.2 v/v% in controls and in flavonoid‐containing incubates.

Flavonoids did not affect CYP2C9‐catalyzed diclofenac hydroxylation (Figure 4A), and only AMP showed statistically significant (*p* < .01) but minor (5%) inhibitory action on CYP2C19‐mediated (*S*)‐mephenytoin hydroxylation (Figure 4B). CYP3A4‐catalyzed testosterone hydroxylation was inhibited by each flavonoid tested (Figure 4C); however, we noticed less than 10% decrease in metabolite formation. Sulfate derivatives showed similar or slightly weaker effects on CYPs compared to their parent flavonoids.

### 3.3 Interaction of MYR, M3′S, AMP, and A4′S with OATP transporters

**FIGURE 5:**
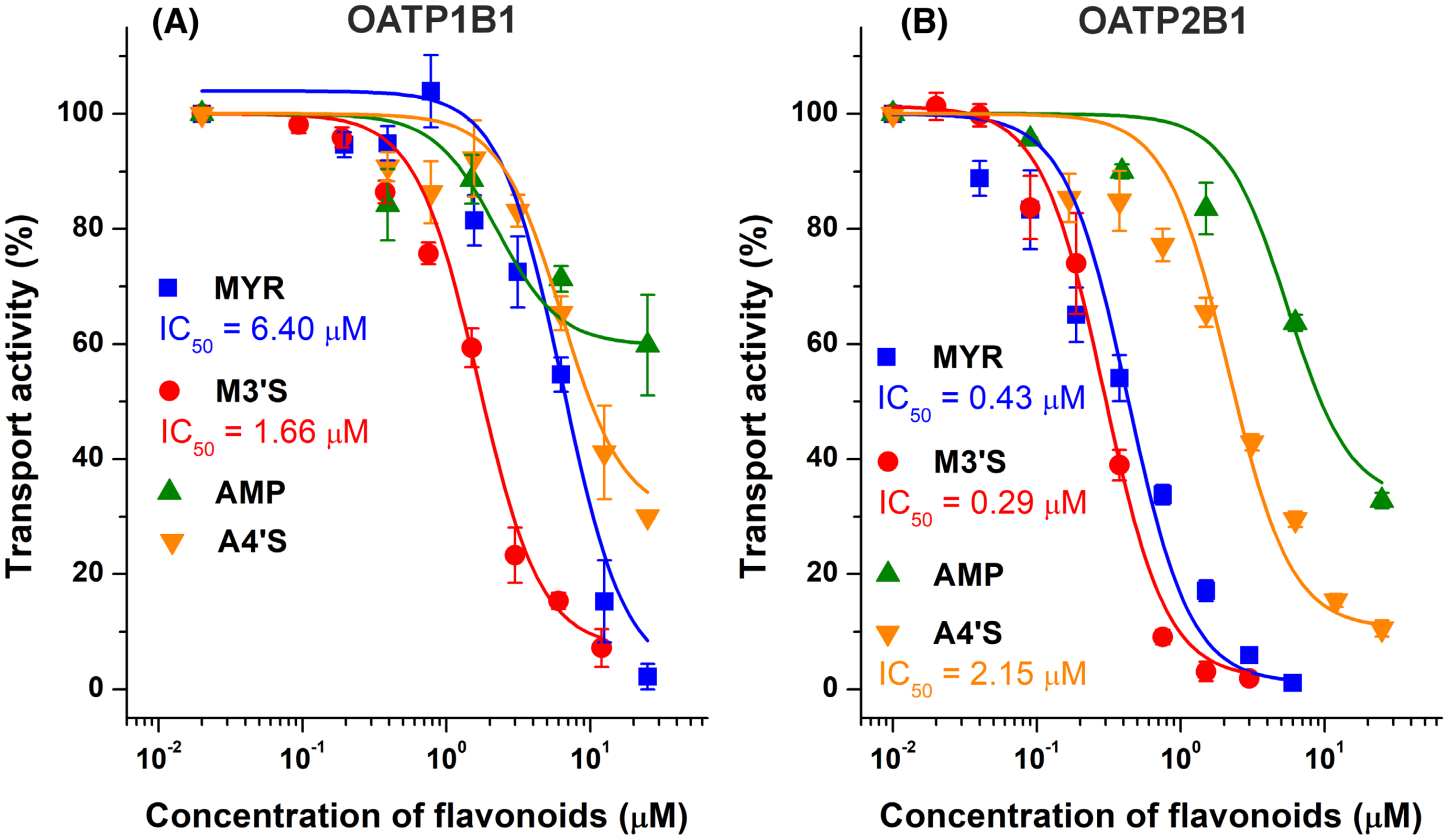
Concentration‐dependent inhibitory effects of MYR (blue), M3′S (red), AMP (green), and A4′S (orange) on OATP1B1 (A) and OATP2B1 (B). The transport of disulfopyranine (OATP1B1) and pyranine (OATP2B1) in A431 cells was evaluated in the absence or presence of increasing concentrations of flavonoids (0–25 μM; see further experimental details in Section 2.6). The mean (% of control ± SEM) values are demonstrated (*n* = 3), where the fluorescence measured in the absence of flavonoids was set as 100%.

First, we examined the potential inhibitory actions of MYR, M3′S, AMP, and A4′S on OATP1B1 and OATP2B1 activity. In the concentration range (0–25 μM) tested, each flavonoid exerted a statistically significant (*p* < .01) inhibitory effect on both OATP1B1 and OATP2B1 (Figure 5). At 25 μM concentration, AMP and A4′S induced approximately 40% and 70% decrease in the transport activity of OATP1B1, while the same levels of MYR and M3′S caused close to complete inhibition of this transporter (Figure 5A). Regarding OATP1B1, the IC_50_ value of MYR was 6.4 μM, and its sulfate conjugate M3′S proved to be a four‐fold stronger inhibitor (IC_50_ = 1.7 μM).

In OATP2B1 overexpressing cells, AMP showed again the weakest impact, inducing approximately 70% inhibition at 25 μM concentration (Figure 5B). The same level of A4′S caused a 90% reduction in transport activity; in addition, MYR and M3′S almost completely blocked the OATP2B1‐mediated transport. The IC_50_ value of A4′S was 2.2 μM, while MYR (IC_50_ = 0.4 μM) and M3′S (IC_50_ = 0.3 μM) showed five‐fold and seven‐fold stronger inhibitory effects than A4′S, respectively.

**FIGURE 6:**
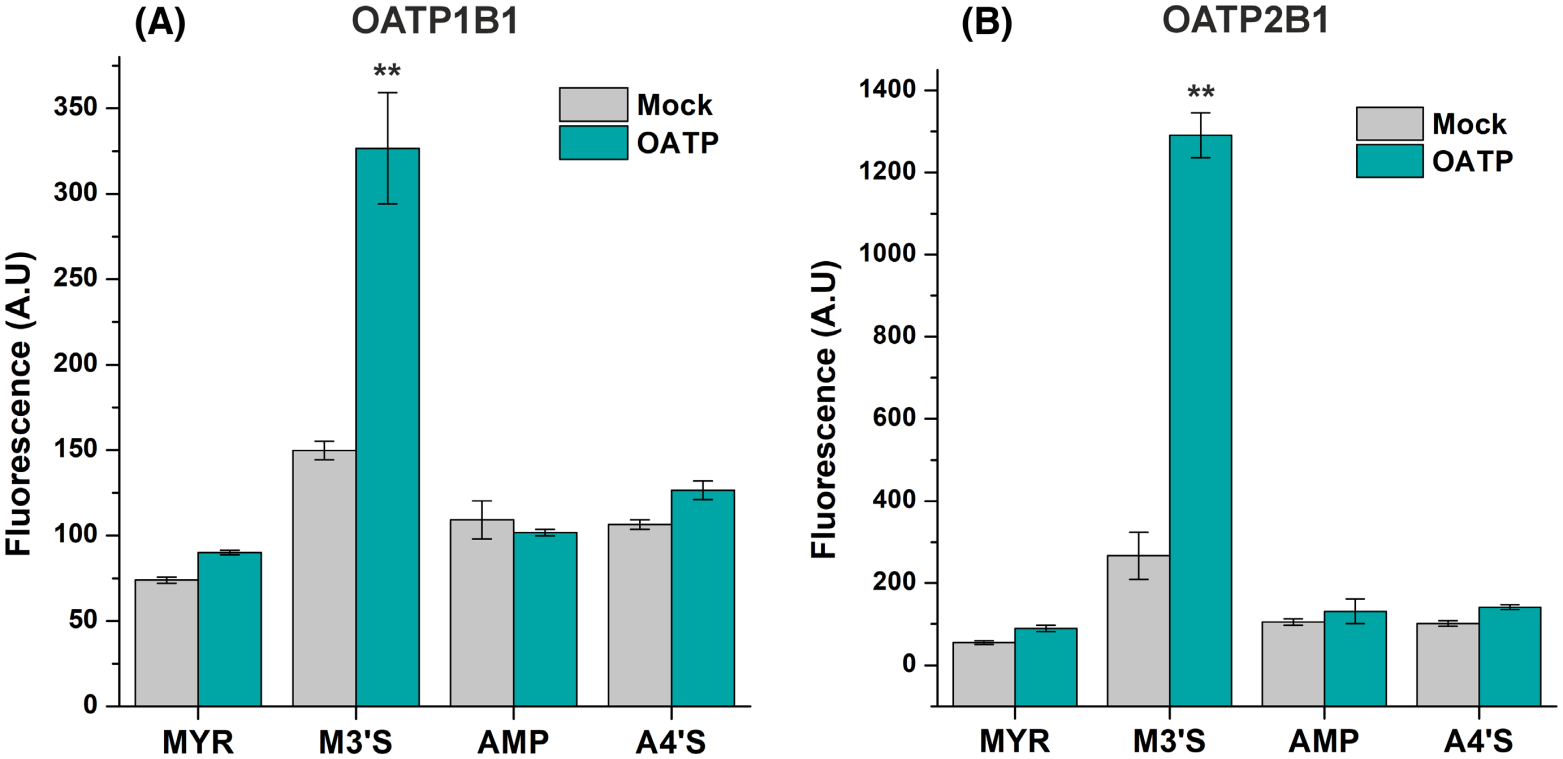
The cellular uptake of MYR, M3′S, AMP, and A4′S in A431 cells overexpressing OATP1B1 (A) or OATP2B1 (B) was determined by exposing the cells to flavonoids (10 μM) for 15 min. Flavonoids were visualized by adding APB + BSA (see experimental details in Section 2.8). The mean (± SEM) values are demonstrated (*n* = 3; **p < .01).

To test the potential OATP‐mediated uptake of MYR, M3′S, AMP, or A4′S, our recently reported method was used. APB is a small fluorescence enhancer that can form a highly fluorescent complex with certain flavonoids, allowing flavonoids' detection based on fluorescence measurement in living cells. After we confirmed that APB can increase the fluorescence signals of MYR, M3′S, AMP, and A4′S in a cell‐free environment, OATP‐overexpressing cells and their mock controls were tested for the uptake of these flavonoids. Cellular concentrations of MYR, AMP, and A4′S did not show statistically significant differences (*p* < .05) in the OATP‐overexpressing versus their mock control cells (Figure 6). However, compared to the mock controls, M3′S levels were more than two‐fold and almost five‐fold higher in OATP1B1‐ (Figure 6A) and OATP2B1‐overexpressing cells (Figure 6B), respectively.

**FIGURE 7:**
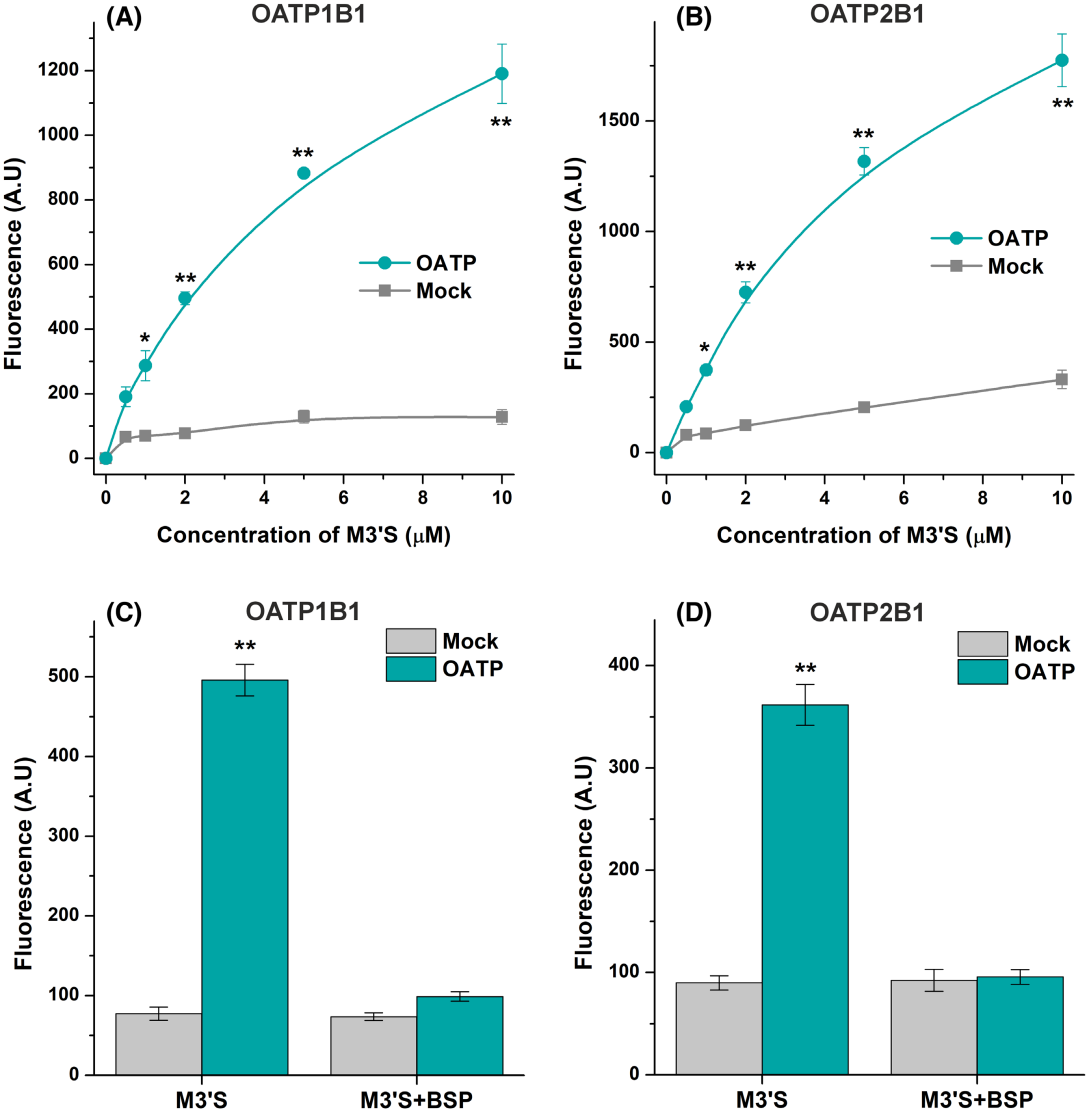
Concentration‐dependent uptake of M3′S by A431 cells overexpressing OATP1B1 (A) or OATP2B1 (B) versus their mock‐transfected controls, and the effects of BSP (OATP inhibitor) on the uptake of M3′S by OATP1B1 (C) and OATP2B1 (D). Cells were incubated with M3′S for 2 min (OATP1B1; A) or 5 min (OATP2B1; B). The impacts of BSP on M3′S (2 μM) transport in A431‐OATP1B1 and A431‐OATP2B1 cells (and their mock controls) were determined after the incubation with or without BSP (20 μM) for 5 min (C, D). Cells were rinsed, then the fluorescence was determined after adding APB + BSA (see further experimental details in Section 2.8). The mean (± SEM) values demonstrated were obtained from three biological replicates (*n* = 3; *p < .05, **p < .01).

In the next step, the OATP‐mediated uptake of M3′S was examined in the 0–10 μM concentration range. The results demonstrate a saturable uptake of M3′S in OATP1B1‐ and OATP2B1‐overexpressing cells, while much slower and close to the linear elevation of cellular M3′S levels were noticed in the mock control cells (Figure 7A,B).

Finally, we also investigated the impacts of BSP (a known inhibitor of OATP1B1 and OATP2B1) on the uptake of M3′S. In the mock cells, BSP did not affect the cellular concentrations of M3′S. However, BSP considerably decreased the uptake of M3′S into OATP1B1‐ and OATP2B1‐overexpressing cells, resulting in similarly low cellular levels of the flavonoid as in the mock control cells (Figure 7C,D).

## 4 DISCUSSION

The total dietary flavonol intake is estimated between 20 and 30 mg/day, which includes only 1–3 mg/day of MYR (while we did not find data regarding AMP).^48^, ^49^, ^50^ Certain dietary supplements contain large doses of MYR and AMP (typical recommended daily doses: 100–600 mg), which highly exceeds the nutritional intake.^2^ The simultaneous administration of these supplements with drugs may negatively influence pharmacotherapy, where not only the parent aglycones but their metabolites may also be involved. Therefore, in the current explorative study, we aimed to examine the interactions of MYR, M3′S, AMP, and A4′S with HSA, CYPs, and OATPs by applying various in vitro assays.

Trp‐214 is the sole tryptophan constituent in HSA, its fluorescence is sensitive to microenvironmental changes.^12^, ^39^ The complex formation of a ligand molecule with albumin typically decreases the emission signal of Trp‐214.^17^, ^18^ Importantly, flavonoids did not exert background fluorescence at 340 nm, and their inner‐filter effects were corrected before evaluation. We noticed the flavonoid‐induced, gradual decreases in the emission signal of the protein at 340 nm (Figure 2), suggesting the formation of flavonoid–HSA complexes. *K* values of M3′S and MYR exceeded 10^5^ L/mol, representing their strong interactions with HSA. However, AMP and A4′S (*K* ≈ 10^4^ L/mol) showed moderate affinity toward the protein. Our results are in good agreement with the previously reported data regarding MYR–HSA, and AMP–HSA complexes.^14^, ^15^

Since only free unbound warfarin can pass through the filter applied, the elevated levels of warfarin in the filtrate indicate the decreased albumin‐bound fraction of the site marker.^17^, ^18^ We selected phenylbutazone as a positive control because earlier studies demonstrated its strong interaction with the Site I region of HSA: its bound concentration in the plasma is approximately 98%–99%, its binding constant is around 10^6^ L/mol, and it can effectively displace warfarin from HSA.^13^, ^51^, ^52^ In ultrafiltration studies, AMP and A4′S did not affect while M3′S and MYR only moderately increased the filtered fraction of warfarin (Figure 3). Thus, ultrafiltration experiments confirmed the stronger interactions of M3′S and MYR with HSA compared to AMP and A4′S. The higher binding affinity and the stronger displacing effect of M3′S versus MYR are in accordance with earlier studies, where the sulfate conjugation of quercetin, chrysin, luteolin, and naringenin also improved the albumin binding of the parent flavonoids.^16^, ^17^, ^18^ However, as an opposite example, quenching studies demonstrated the lower stability of A4′S–HSA versus AMP–HSA complex (Figure 2). Importantly, M3′S and MYR showed statistically significant, although considerably weaker displacing effects compared to the positive control phenylbutazone (Figure 3). In addition, under the same experimental conditions, quercetin‐3′‐sulfate and chrysin‐7‐sulfate almost completely displaced the site marker,^17^, ^18^ which underlines again the limited displacing ability of M3′S and MYR. Therefore, it is very unlikely that MYR, M3′S, AMP, or A4′S could affect the albumin binding of warfarin or other Site I ligand drugs under clinical conditions, and these results presumably have only some theoretical importance.

Previous in vitro investigations performed using human or rat liver microsomes suggest the weak to moderate inhibitory effects of MYR on CYP2C9 and CYP3A4, and that of AMP on CYP3A4.^21^, ^22^, ^23^, ^24^ In the current study, we noticed no or only slight decreases in CYP2C9, CYP2C19, and CYP3A4 activity in the presence of MYR, AMP, or their sulfate conjugates (Figure 4). Importantly, we tested the impacts of 20 μM flavonoid concentrations, which highly exceed their possible peak plasma levels. In a rat experiment, MYR increased the plasma levels of tolbutamide (a substrate of CYP2C9) and midazolam (a substrate of CYP3A4).^53^ In another rat study, the peroral co‐administration of MYR with docetaxel (a substrate of CYP3A4) increased the oral bioavailability and peak plasma concentration of the drug.^54^ However, it has also been demonstrated that the inhibitory impact of MYR on CYP3A4 has no significant in vivo relevance, while the elevated oral bioavailability of docetaxel is likely caused by the MYR‐induced inhibition of P‐glycoprotein.^54^ Considering the above‐listed observations, it seems to be unlikely that MYR, AMP, and/or their sulfate metabolites could strongly affect the CYP‐mediated elimination of drugs.

We noticed a weak inhibitory action of AMP on OATP1B1 (Figure 5A), which is coherent with the previous observations.^28^ Furthermore, our results suggest for the first time, that AMP is also a weak inhibitor of OATP2B1 (Figure 5B). Importantly, in the same experimental model, the positive control inhibitor bromosulfophthalein showed 0.06 μM and 1.3 μM IC_50_ values regarding OATP1B1 and OATP2B1 transporters, respectively.^33^ In our study, MYR showed a moderate inhibitory effect (IC_50_ = 6.4 μM) on OATP1B1‐mediated disulfopyrene uptake (Figure 5A). Very similar inhibitory action of MYR (IC_50_ = 5.8 μM) has been reported for 2′,7′‐dichlorofluorescein uptake,^31^ while 50 μM concentration of MYR resulted in approximately 35% decrease in the OATP1B1‐mediated transport of dehydroepiandrosterone sulfate and fluvastatin.^28^, ^30^ In the current work, MYR was a highly potent inhibitor (IC_50_ = 0.4 μM) of OATP2B1‐mediated pyranine uptake (Figure 5B). However, previous reports described only weak inhibitory effects of MYR on OATP2B1‐dependent transport of 4′,5′‐dibromofluorescein and estrone 3‐sulfate.^29^, ^32^ These differences may suggest some substrate specificity regarding the inhibitory potency of MYR on OATP1B1 and OATP2B1, which has been previously demonstrated regarding certain other OATP inhibitors.^44^, ^55^

To the best of our knowledge, this is the first study that examined the interactions of M3′S and A4′S with OATPs. Both sulfate conjugates proved to be stronger inhibitors of OATP1B1 and OATP2B1 than their parent aglycones (Figure 5). In line with these findings, sulfate conjugates of chrysin, quercetin, luteolin, and naringenin showed similar or stronger inhibitory effects on OATP1B1 and OATP2B1 compared to their aglycones.^16^, ^33^, ^34^ Since sulfate and glucuronic acid conjugates are the dominant forms of flavonoids in circulation, the potent inhibitory actions of flavonoid sulfates on certain OATPs may have high pharmacological importance. A4′S can be considered as a weak inhibitor of OATP1B1 (Figure 5). However, we observed low micromolar IC_50_ values of M3′S (IC_50_ = 1.7 μM) and A4′S (IC_50_ = 2.2 μM) regarding OATP1B1 and OATP2B1, respectively. In addition, even nanomolar concentrations of M3′S (IC_50_ = 0.3 μM) caused remarkable decreases in OATP2B1 activity. Unfortunately, no data are available regarding the plasma levels of M3′S and A4′S. However, based on human studies with quercetin (which has a similar chemical structure),^56^, ^57^, ^58^ it is reasonable to hypothesize the high nanomolar or low micromolar peak plasma concentrations of M3′S and A4′S as a result of the repeated administration of MYR‐ and AMP‐containing dietary supplements (with hundreds of mg daily doses of MYR/AMP), respectively. Therefore, our data suggest that the high intake of MYR and AMP may affect the OATP‐driven uptake of certain drugs, likely with the significant involvement of sulfate metabolites. In addition, the consumption of dietary supplements can result in high levels of MYR in the gastrointestinal tract, which may influence the OATP2B1‐mediated absorption of some medications.

We also demonstrated that the presence of OATP1B1 or OATP2B1 does not affect the cellular levels of MYR, AMP, and A4′S (Figure 6); thus, these flavonoids are inhibitors but not substrates of the OATPs examined. However, considerably higher concentrations of M3′S were observed in OATP‐overexpressing versus mock control cells (Figure 6). In addition, the saturable concentration‐dependent uptake of M3′S as well as the inhibitory effect of BSP on the OATP‐mediated uptake of M3′S were confirmed (Figure 7). These findings prove that M3′S is a substrate of both OATP1B1 and OATP2B1. Thus, M3′S can be taken up by OATP1B1‐ and OATP2B1‐expressing cells through these carrier‐mediated transport mechanisms. Nevertheless, it is important to note that the strong albumin binding of M3′S may limit its interaction (inhibition/uptake) with OATPs.

In conclusion, the interactions of MYR, M3′S, AMP, and A4′S were examined with HSA, CYPs, and OATPs applying in vitro models. M3′S and MYR showed strong interactions with HSA, and they moderately displaced the Site I marker warfarin from the protein; while AMP and A4′S bound to albumin with lower affinity. The flavonoids assessed caused no or only slight inhibitory effects on CYP2C9, CYP2C19, and CYP3A4 activity. M3′S and MYR caused a strong decrease in OATP1B1 activity even at a few micromolar concentrations. Furthermore, M3′S and MYR were highly potent inhibitors of OATP2B1 with nanomolar IC_50_ values, and A4′S showed inhibitory action at low micromolar levels. Among the flavonoids examined, only M3′S proved to be a substrate of OATP1B1 and OATP2B1. Considering the above‐listed observations, it is unlikely that these flavonoids can affect the albumin binding and the CYP2C9‐, 2C19‐, or 3A4‐catalyzed biotransformation of drugs. However, our novel observations highlight that M3′S may interfere with the OATP1B1/2B1‐mediated cellular uptake of certain drugs. In addition, the OATP2B1‐dependent intestinal absorption of some medications may also be reduced by MYR. Nevertheless, further in vivo studies are required to confirm the potential clinical relevance of these interactions.

## AUTHOR CONTRIBUTIONS

*Participated in research design*: Miklós Poór and Csilla Özvegy‐Laczka. *Conducted experiments*: Ágnes Dombi, Hana Kaci, and Éva Bakos. *Contributed new reagents or analytic tools*: Kateřina Valentová, Miklós Poór, and Csilla Özvegy‐Laczka. *Performed data analysis*: Miklós Poór, Ágnes Dombi, Hana Kaci, and Éva Bakos. *Wrote or contributed to the writing of the manuscript*: Miklós Poór, Ágnes Dombi, Hana Kaci, Kateřina Valentová, and Csilla Özvegy‐Laczka.

## FUNDING INFORMATION

The work of M.P. is supported by the Hungarian National Research, Development and Innovation Office (FK138184) and by the János Bolyai Research Scholarship of the Hungarian Academy of Sciences (BO/00381/21). The work of Á.D. is supported by the ÚNKP‐23‐4 New National Excellence Program of the Ministry for Culture and Innovation from the source of the National Research, Development and Innovation Fund. The work of Cs. Ö‐L. is supported by the Hungarian National Research, Development, and Innovation Office (K138518). K.V. was supported by the Czech Science Foundation (23‐04654S).

## CONFLICT OF INTEREST STATEMENT

The authors declare that they have no known competing financial interests or personal relationships that could have appeared to influence the work reported in this paper.

## ETHICS STATEMENT

None. No animal experiments or human studies were involved in the current investigation.

## Data Availability

The authors declare that all the data supporting the findings of this study are contained within the paper. The raw data are available upon request from the corresponding author.

## References

[1] Song X , Tan L , Wang M , et al. Myricetin: a review of the most recent research. Biomed Pharmacother. 2021;134:111017. doi:10.1016/j.biopha.2020.111017 33338751

[2] Balázs O , Dombi Á , Zsidó BZ , et al. Inhibition of xanthine oxidase‐catalyzed xanthine and 6‐mercaptopurine oxidation by luteolin, naringenin, myricetin, ampelopsin and their conjugated metabolites. Biomed Pharmacother. 2023;167:115548. doi:10.1016/j.biopha.2023.115548 37734263

[3] Imran M , Saeed F , Hussain G , et al. Myricetin: a comprehensive review on its biological potentials. Food Sci Nutr. 2021;9:5854‐5868. doi:10.1002/fsn3.2513 34646551 PMC8498061

[4] Dang Y , Lin G , Xie Y , et al. Quantitative determination of myricetin in rat plasma by ultra performance liquid chromatography tandem mass spectrometry and its absolute bioavailability. Drug Res. 2014;64:516‐522. doi:10.1055/s-0033-1363220 24357136

[5] Chen J , Wang X , Xia T , et al. Molecular mechanisms and therapeutic implications of dihydromyricetin in liver disease. Biomed Pharmacother. 2021;142:111927. doi:10.1016/j.biopha.2021.111927 34339914

[6] Zhang J , Chen Y , Luo H , et al. Recent update on the pharmacological effects and mechanisms of dihydromyricetin. Front Pharmacol. 2018;9:1204. doi:10.3389/fphar.2018.01204 30410442 PMC6209623

[7] Chen S , Zhao X , Wan J , et al. Dihydromyricetin improves glucose and lipid metabolism and exerts anti‐inflammatory effects in nonalcoholic fatty liver disease: a randomized controlled trial. Pharmacol Res. 2015;99:74‐81. doi:10.1016/j.phrs.2015.05.009 26032587

[8] Fan L , Tong Q , Dong W , et al. Tissue distribution, excretion, and metabolic profile of dihydromyricetin, a flavonoid from vine tea (Ampelopsis grossedentata) after oral administration in rats. J Agric Food Chem. 2017;65:4597‐4604. doi:10.1021/acs.jafc.7b01155 28534405

[9] Liu L , Sun S , Rui H , Li X . In vitro inhibitory effects of dihydromyricetin on human liver cytochrome P450 enzymes. Pharm Biol. 2017a;55:1868‐1874. doi:10.1080/13880209.2017.1339284 28614988 PMC7012011

[10] Tong Q , Hou X , Fang J , et al. Determination of dihydromyricetin in rat plasma by LC–MS/MS and its application to a pharmacokinetic study. J Pharm Biomed Anal. 2015;114:455‐461. doi:10.1016/j.jpba.2015.06.030 26133104

[11] Zhou J , Zeng P , Tu HH , Wang FQ . Development and application of high‐performance liquid chromatography for the study of ampelopsin pharmacokinetics in rat plasma using cloud‐point extraction. J Sep Sci. 2011;34:160‐168. doi:10.1002/jssc.201000382 21246721

[12] Fanali G , di Masi A , Trezza V , Marino M , Fasano M , Ascenzi P . Human serum albumin: from bench to bedside. Mol Asp Med. 2012;33:209‐290. doi:10.1016/j.mam.2011.12.002 22230555

[13] Yamasaki K , Chuang VTG , Maruyama T , Otagiri M . Albumin‐drug interaction and its clinical implication. Biochim Biophys Acta. 2013;1830:5435‐5443. doi:10.1016/j.bbagen.2013.05.005 23665585

[14] Chen T , Zhu S , Lu Y , et al. Probing the interaction of anti‐cancer agent dihydromyricetin with human serum albumin: a typical method study. Anti Cancer Agents Med Chem. 2012;12:919‐928. doi:10.2174/187152012802650002 22292768

[15] Qin C , Xie MX , Liu Y . Characterization of the myricetin‐human serum albumin complex by spectroscopic and molecular modeling approaches. Biomacromolecules. 2007;8:2182‐2189. doi:10.1021/bm070319c 17559264

[16] Kaci H , Bodnárová S , Fliszár‐Nyúl E , et al. Interaction of luteolin, naringenin, and their sulfate and glucuronide conjugates with human serum albumin, cytochrome P450 (CYP2C9, CYP2C19, and CYP3A4) enzymes and organic anion transporting polypeptide (OATP1B1 and OATP2B1) transporters. Biomed Pharmacother. 2023;157:114078. doi:10.1016/j.biopha.2022.114078 36481402

[17] Mohos V , Fliszár‐Nyúl E , Schilli G , et al. Interaction of chrysin and its main conjugated metabolites chrysin‐7‐sulfate and chrysin‐7‐glucuronide with serum albumin. Int J Mol Sci. 2018;19:4073. doi:10.3390/ijms19124073 30562928 PMC6320863

[18] Poór M , Boda G , Needs PW , Kroon PA , Lemli B , Bencsik T . Interaction of quercetin and its metabolites with warfarin: displacement of warfarin from serum albumin and inhibition of CYP2C9 enzyme. Biomed Pharmacother. 2017;88:574‐581. doi:10.1016/j.biopha.2017.01.092 28135601

[19] McDonnell AM , Dang CH . Basic review of the cytochrome P450 system. J Adv Pract Oncol. 2013;4:263‐268. doi:10.6004/jadpro.2013.4.4.7 25032007 PMC4093435

[20] Zhao M , Ma J , Li M , et al. Cytochrome P450 enzymes and drug metabolism in humans. Int J Mol Sci. 2021;22:12808. doi:10.3390/ijms222312808 34884615 PMC8657965

[21] Li Y , Ning J , Wang Y , et al. Drug interaction study of flavonoids toward CYP3A4 and their quantitative structure activity relationship (QSAR) analysis for predicting potential effects. Toxicol Lett. 2018;294:27‐36. doi:10.1016/j.toxlet.2018.05.008 29753067

[22] Liu L , Yin X , Wang X , Li X . Determination of dihydromyricetin in rat plasma by LC‐MS/MS and its application to a pharmacokinetic study. Pharm Biol. 2017b;55:657‐662. doi:10.1080/13880209.2016.1266669 27951743 PMC6130699

[23] Lou D , Bao SS , Li YH , Lin QM , Yang SF , He JY . Inhibitory mechanisms of myricetin on human and rat liver cytochrome P450 enzymes. Eur J Drug Metab Pharmacokinet. 2019;44:611‐618. doi:10.1007/s13318-019-00546-y 30825074

[24] Östlund J , Zlabek V , Zamaratskaia G . In vitro inhibition of human CYP2E1 and CYP3A by quercetin and myricetin in hepatic microsomes is not gender dependent. Toxicology. 2017;381:10‐18. doi:10.1016/j.tox.2017.02.012 28232125

[25] Shitara Y , Maeda K , Ikejiri K , Yoshida K , Horie T , Sugiyama Y . Clinical significance of organic anion transporting polypeptides (OATPs) in drug disposition: their roles in hepatic clearance and intestinal absorption: clinical significance of OATPs in drug disposition. Biopharm Drug Dispos. 2013;34:45‐78. doi:10.1002/bdd.1823 23115084

[26] Kinzi J , Grube M , Meyer Zu Schwabedissen HE . OATP2B1—the underrated member of the organic anion transporting polypeptide family of drug transporters? Biochem Pharmacol. 2021;188:114534. doi:10.1016/j.bcp.2021.114534 33794186

[27] Shitara Y . Clinical importance of OATP1B1 and OATP1B3 in drug‐drug interactions. Drug Metab Pharmacokinet. 2011;26:220‐227. doi:10.2133/dmpk.DMPK-10-RV-094 21297316

[28] Fan X , Bai J , Hu M , et al. Drug interaction study of flavonoids toward OATP1B1 and their 3D structure activity relationship analysis for predicting hepatoprotective effects. Toxicology. 2020;437:152445. doi:10.1016/j.tox.2020.152445 32259555

[29] Peng T , Liu S , Li Y , Zhang H , Hagenbuch B , Gui C . Investigating the interactions of flavonoids with human OATP2B1: inhibition assay, IC50 determination, and structure‐activity relationship analysis. RSC Med Chem. 2023;14:890‐898. doi:10.1039/d3md00078h 37252098 PMC10211325

[30] Wang X , Wolkoff AW , Morris ME . Flavonoids as a novel class of human organic anion‐transporting polypeptide OATP1B1 (OATP‐C) modulators. Drug Metab Dispos. 2005;33:1666‐1672. doi:10.1124/dmd.105.005926 16081670

[31] Xiang Y , Liu S , Yang J , Wang Z , Zhang H , Gui C . Investigation of the interactions between flavonoids and human organic anion transporting polypeptide 1B1 using fluorescent substrate and 3D‐QSAR analysis. Biochim Biophys Acta Biomembr. 2020;1862:183210. doi:10.1016/j.bbamem.2020.183210 32006472

[32] Navrátilová L , Mandíková JR , Pávek P , Mladěnka P , Trejtnar F . Honey flavonoids inhibit hOATP2B1 and hOATP1A2 transporters and hOATP‐mediated rosuvastatin cell uptake in vitro. Xenobiotica. 2018;48:745‐755. doi:10.1080/00498254.2017.1358469 28745105

[33] Mohos V , Fliszár‐Nyúl E , Ungvári O , et al. Effects of chrysin and its major conjugated metabolites chrysin‐7‐sulfate and chrysin‐7‐glucuronide on cytochrome P450 enzymes and on OATP, P‐gp, BCRP, and MRP2 transporters. Drug Metab Dispos. 2020a;48:1064‐1073. doi:10.1124/dmd.120.000085 32661014

[34] Mohos V , Fliszár‐Nyúl E , Ungvári O , et al. Inhibitory effects of quercetin and its main methyl, sulfate, and glucuronic acid conjugates on cytochrome P450 enzymes, and on OATP, BCRP and MRP2 transporters. Nutrients. 2020b;12:2306. doi:10.3390/nu12082306 32751996 PMC7468908

[35] Kaci H , Bakos É , Needs PW , et al. The 2‐aminoethyl diphenylborinate‐based fluorescent method identifies quercetin and luteolin metabolites as substrates of organic anion transporting polypeptides, OATP1B1 and OATP2B1. Eur J Pharm Sci. 2024;196:106740. doi:10.1016/j.ejps.2024.106740 38437885

[36] Káňová K , Petrásková L , Pelantová H , et al. Sulfated metabolites of luteolin, myricetin, and ampelopsin: Chemoenzymatic preparation and biophysical properties. J Agric Food Chem. 2020;68:11197‐11206. doi:10.1021/acs.jafc.0c03997 32910657

[37] Hu T , Liu Y . Probing the interaction of cefodizime with human serum albumin using multi‐spectroscopic and molecular docking techniques. J Pharm Biomed Anal. 2015;107:325‐332. doi:10.1016/j.jpba.2015.01.010 25637820

[38] Mohos V , Fliszár‐Nyúl E , Lemli B , et al. Testing the pharmacokinetic interactions of 24 colonic flavonoid metabolites with human serum albumin and cytochrome P450 enzymes. Biomol Ther. 2020c;10:409. doi:10.3390/biom10030409 PMC7175153 32155912

[39] van de Weert M , Stella L . Fluorescence quenching and ligand binding: a critical discussion of a popular methodology. J Mol Struct. 2011;998:144‐150. doi:10.1016/j.molstruc.2011.05.023

[40] Mohammadgholi A , Leilabadi‐Asl A , Divsalar A , Eslami‐Moghadam M . Multi‐spectroscopic studies of the interaction of new synthesized platin complex with human carrier protein of serum albumin. J Biomol Struct Dyn. 2021;39:1506‐1511. doi:10.1080/07391102.2020.1745690 32200700

[41] Peschke M , Verkerk UH , Kebarle P . Features of the ESI mechanism that affect the observation of multiply charged noncovalent protein complexes and the determination of the association constant by the titration method. J Am Soc Mass Spectrom. 2004;15:1424‐1434. doi:10.1016/j.jasms.2004.05.005 15465355

[42] Bakos É , Német O , Patik I , et al. A novel fluorescence‐based functional assay for human OATP1A2 and OATP1C1 identifies interaction between third‐generation P‐gp inhibitors and OATP1A2. FEBS J. 2020;287:2468‐2485. doi:10.1111/febs.15156 31770475

[43] Patik I , Székely V , Német O , et al. Identification of novel cell‐impermeant fluorescent substrates for testing the function and drug interaction of organic anion‐transporting polypeptides, OATP1B1/1B3 and 2B1. Sci Rep. 2018;8:2630. doi:10.1038/s41598-018-20815-1 29422623 PMC5805760

[44] Izumi S , Nozaki Y , Komori T , et al. Substrate‐dependent inhibition of organic anion transporting polypeptide 1B1: comparative analysis with prototypical probe substrates estradiol‐17β‐glucuronide, estrone‐3‐sulfate, and sulfobromophthalein. Drug Metab Dispos. 2013;41:1859‐1866. doi:10.1124/dmd.113.052290 23920221

[45] Harding SD , Sharman JL , Faccenda E , et al. The IUPHAR/BPS guide to PHARMACOLOGY in 2019: updates and expansion to encompass the new guide to IMMUNOPHARMACOLOGY. Nucleic Acids Res. 2018;46:D1091‐D1106. doi:10.1093/nar/gkx1121 29149325 PMC5753190

[46] Alexander SPH , Fabbro D , Kelly E , et al. The concise guide to PHARMACOLOGY 2023/24: enzymes. Br J Pharmacol. 2023a;180:S289‐S373. doi:10.1111/bph.16181 38123154

[47] Alexander SPH , Fabbro D , Kelly E , et al. The concise guide to PHARMACOLOGY 2023/24: transporters. Br J Pharmacol. 2023b;180:S374‐S469. doi:10.1111/bph.16182 38123156

[48] Mullie P , Clarys P , Deriemaeker P , Hebbelinck M . Estimation of daily human intake of food flavonoids. Plant Foods Hum Nutr. 2007;62:93‐98. doi:10.1007/s11130-007-0047-7 17597415

[49] Murphy KJ , Walker KM , Dyer KA , Bryan J . Estimation of daily intake of flavonoids and major food sources in middle‐aged Australian men and women. Nutr Res. 2019;61:64‐81. doi:10.1016/j.nutres.2018.10.006 30683440

[50] Zamora‐Ros R , Andres‐Lacueva C , Lamuela‐Raventós RM , et al. Estimation of dietary sources and flavonoid intake in a Spanish adult population (EPIC‐Spain). J Am Diet Assoc. 2010;110:390‐398. doi:10.1016/j.jada.2009.11.024 20184989

[51] Aarbakke J . Clinical pharmacokinetics of phenylbutazone. Clin Pharmacokinet. 1978;3:369‐380. doi:10.2165/00003088-197803050-00003 359213

[52] Veronich K , White G , Kapoor A . Effects of phenylbutazone, tolbutamide, and clofibric acid on binding of racemic warfarin and its enantiomers to human serum albumin. J Pharm Sci. 1979;68:1515‐1518. doi:10.1002/jps.2600681213 529041

[53] Guo YJ , Zheng SL . Effect of myricetin on cytochrome P450 isoforms CYP1A2, CYP2C9 and CYP3A4 in rats. Pharmazie. 2014;69:306‐310. doi:10.1691/ph.2014.3842 24791597

[54] Hao T , Ling Y , Wu M , et al. Enhanced oral bioavailability of docetaxel in rats combined with myricetin: in situ and in vivo evidences. Eur J Pharm Sci. 2017;101:71‐79. doi:10.1016/j.ejps.2017.02.009 28185989

[55] Noé J , Portmann R , Brun M‐E , Funk C . Substrate‐dependent drug‐drug interactions between gemfibrozil, fluvastatin and other organic anion‐transporting peptide (OATP) substrates on OATP1B1, OATP2B1, and OATP1B3. Drug Metab Dispos. 2007;35:1308‐1314. doi:10.1124/dmd.106.012930 17470528

[56] Cialdella‐Kam L , Nieman DC , Sha W , Meaney MP , Knab AM , Shanely RA . Dose‐response to 3 months of quercetin‐containing supplements on metabolite and quercetin conjugate profile in adults. Br J Nutr. 2013;109:1923‐1933. doi:10.1017/S0007114512003972 23151341

[57] Day AJ , Mellon F , Barron D , Sarrazin G , Morgan MR , Williamson G . Human metabolism of dietary flavonoids: identification of plasma metabolites of quercetin. Free Radic Res. 2001;35:941‐952. doi:10.1080/10715760100301441 11811545

[58] Mullen W , Edwards CA , Crozier A . Absorption, excretion and metabolite profiling of methyl‐, glucuronyl‐, glucosyl‐ and sulpho‐conjugates of quercetin in human plasma and urine after ingestion of onions. Br J Nutr. 2006;96:107‐116. doi:10.1079/bjn20061809 16869998

